# A chronometric relationship between circuits underlying learning and error monitoring in the basal ganglia and salience network

**DOI:** 10.1162/imag_a_00343

**Published:** 2024-11-05

**Authors:** Camila Dias, Teresa Sousa, Miguel Castelo-Branco

**Affiliations:** CIBIT — Coimbra Institute for Biomedical Imaging and Translational Research, University of Coimbra, Coimbra, Portugal; ICNAS — Institute for Nuclear Sciences Applied to Health, University of Coimbra, Coimbra, Portugal; FMUC — Institute of Physiology, Faculty of Medicine, University of Coimbra, Coimbra, Portugal; LASI — Intelligent Systems Associate Laboratory, Guimarães, Portugal

**Keywords:** error monitoring, learning, dACC, anterior insula, putamen

## Abstract

Healthy individuals readily adjust their behavior in response to errors usinglearning mechanisms. This raises the question of how error-related neuralmechanisms underlie the learning process and its progress. In this study, 21healthy participants performed a challenging functional magnetic resonanceimaging (fMRI) task to answer this question. We assessed the evolution oferror-related neural response as a function of learning progress. We tested thehypothesis that the dorsal anterior cingulate cortex (dACC) and anterior insula,key regions of the error monitoring neural circuitry, reflect both theperformance of an action and its improvement. Given the nature oftrial-and-error learning, we also expected an involvement of the striatum,particularly the putamen. We found that error-related neural activity (in thedACC and anterior insula) was similar following correct responses and errors inan initial learning period. However, as learning progressed, the activitycontinuously decreased in response to correct events and increased after errors.In opposition, during the initial learning phase, the putamen activity wasmodulated by errors, but, as it progressed, this region became unaffected byresponse outcomes. In sum, our study provides neural evidence for an interactionbetween the mechanisms underlying error monitoring and learning, contributing toclarifying how error-related neural responses evolve with learning.

## 1 Introduction

Error monitoring enables one to detect, process, and signal errors prior to, during,and following actions (Ullsperger et al.,2014). It facilitates more efficient actions by helping minimizesubsequent mistakes, which makes it crucial for adaptive behavior and learning(Ninomiya et al., 2018;Ullsperger, 2017;Ullsperger et al., 2014).

Electroencephalographic (EEG) research has identified the error-related negativity(ERN), a midfrontal negative event-related potential (ERP) estimated to originate inthe dorsal anterior cingulate cortex (dACC) (Neta etal., 2015;Ullsperger et al.,2014) that arises 50-100 ms after erroneous actions (Bhattacharyya et al., 2017;Chavarriaga et al., 2014;Estiveira et al., 2022). According to the reinforcement learning theoryof the ERN (Holroyd & Coles, 2002),this ERP reflects reward prediction errors, which are dopaminergic signals thatencode discrepancies between expected and actual outcomes to trigger appropriateadjustments and optimize behavior (Alexander &Brown, 2011;Ullsperger et al.,2014). In line with this, functional magnetic resonance imaging (fMRI)studies link the dACC to an extensive range of functions, including salience,reward-based decision making, conflict, and error monitoring. The dACC has also beenhypothesized to monitor external and internal processes which contribute to makingpredictions about action outcomes and providing action feedback to downstreamcircuits. Such activity is employed to update predictions and optimize futurebehavior. Therefore, it is believed that error and conflict are tracked by the dACCand signaled when additional control and cognitive resources are needed (Heilbronner & Hayden, 2016;Weiss et al., 2018).

Even though most error monitoring studies have focused on the activity of the dACC,several other regions have been linked to error monitoring processes, such as theanterior insula, pre-supplementary area (pre-SMA), basal ganglia, and lateralprefrontal cortex (Neta et al., 2015;Ninomiya et al., 2018;Ullsperger et al., 2014). The anterior insula, inparticular, has been consistently shown to have an important role in errorawareness, as previous studies reported increased activity in perceived compared tounperceived errors (Dali et al., 2023;Harsay et al., 2012;Klein et al., 2007). It has been suggested that the dACCcontributes to error awareness as well (Dali et al.,2023;Orr & Hester, 2012).In accordance with this, both regions have been proposed to be involved ininteroceptive awareness, and, more precisely, to integrate autonomic informationwith salient events such as errors (Dali et al.,2023;Estiveira et al., 2022;Harsay et al., 2012;Klein et al., 2007,^2013^).

The anterior insula and dACC are thus frequently co-activated for a variety ofcognitive processes. These structures have been suggested to form the saliencenetwork, which is vital for monitoring important stimuli that require autonomicregulation (Dali et al., 2023;Harsay et al., 2012;Klein et al., 2013). Once sensory areas recognize asalient event, this information is transmitted to the salience network. Thisnetwork, in turn, triggers a signal to engage brain regions that mediateattentional, working memory, and action selection processes, while disengaging thedefault mode network. Errors can be seen as salient events due to their occasionaloccurrence and usefulness in guiding behavior and learning (Harsay et al., 2012).

Although the neural processes underlying error monitoring have been extensivelystudied, their relationship with learning mechanisms remains unclear.Kennerley et al. (2006)demonstrated that theACC is vital for learning: the authors found that lesions in the ACC lead toimpairments in integrating past feedback information, causing monkeys to use onlythe immediate previous feedback as a guide for subsequent choices. Additionally,previous studies have found that error-related dACC activity predicts post-erroradaptations (Danielmeier et al., 2011) anderror correction (Hester et al., 2008,^2009^). Moreover,Mars et al. (2005)suggested that dACC activity changesduring learning of stimulus-response associations by trial and error. In theirstudy, before learning, error information was not available until the delivery ofexternal performance feedback, but after learning, it was available earlier frominternal sources. Accordingly, the authors reported that error feedback-related dACCactivity decreases as learning proceeds, while error response-related activityincreases. Their results revealed a neural response shift as a function of learning,from external sources provided by error feedback to internal sources linked to theerror response itself. Similarly, using EEG,Bellebaum and Daum (2008)found that feedback-related responses decreasewith learning, while internal error-related responses increase.

Most of these studies used tasks with external performance feedback to analyze theneural bases of learning from errors. Nonetheless, most decisions in daily life arenot followed by feedback. Reward prediction errors are conveyed to the striatum(Ullsperger et al., 2014), andDaniel and Pollmann (2012)demonstrated thatthis dopaminergic region is activated not only by explicit external rewards, such asfood and money, but also by internally generated signals on perceived correctness(Daniel & Pollmann, 2012,^2014^).

In this study, we aimed to clarify how error-related neural mechanisms progress withlearning, and how learning is affected by error monitoring, during a challengingfunctional magnetic resonance imaging (fMRI) task without external feedback. Wehypothesized that the dACC, anterior insula, and striatal regions such as theputamen would play a crucial role in learning from errors. We anticipated that theactivity in these neural regions would be modulated by the relationship betweenerror monitoring and learning. Both error-related brain activity and connectivitywere investigated at different learning periods.

## 2 Methods

### 2.1 Participants

Twenty-one healthy volunteers (11 females, mean age 27.38 ± 5.43 years)participated in this study. Participants with neurological or psychiatricillnesses were excluded, and all participants had normal or corrected-to-normalvisual acuity. All volunteers were right-handed, and the mean laterality indexwas 82.61 ± 20.35 (Oldfield,1971). Every participant completed the magnetic resonance imaging (MRI)safety questionnaires and provided written informed consent in accordance withthe Declaration of Helsinki prior to participation. The study followed thesafety guidelines for research on humans and was approved by the EthicsCommittee of the Faculty of Medicine of the University of Coimbra.

### 2.2 Task

**Fig. 1.**
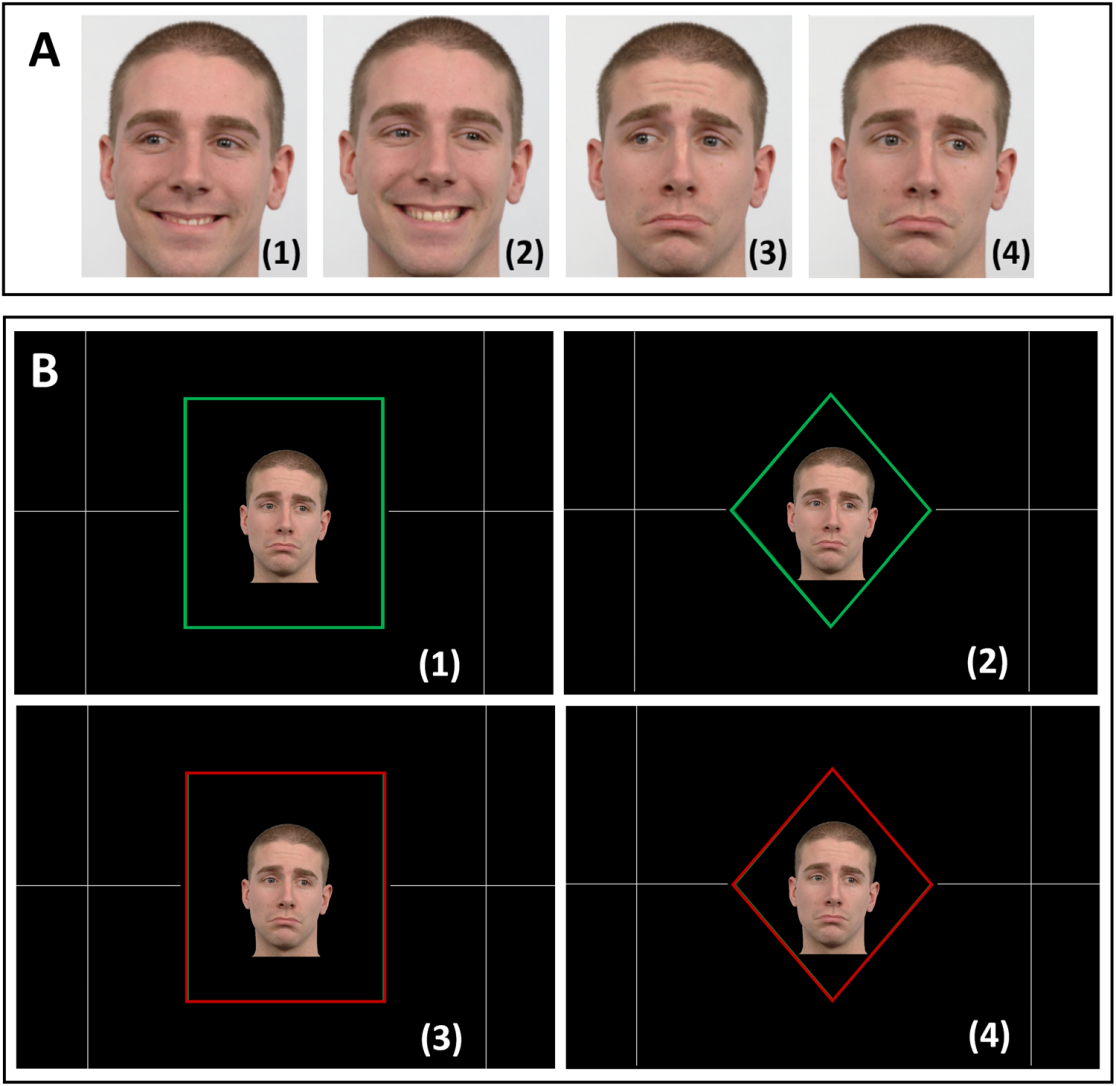
Task instructions. (A) Facial cues: happy left (1), happy right (2), sadleft (3) and sad right (4). (B) Combination of the three cues asinstruction. The frame color was either green (1, 2) or red (3, 4), andthe frame shape could be a rectangle (1, 3) or a diamond (2, 4). If theframe was green, a happy face instructed to act in the same direction ofthe face’s eye gaze, and a sad face instructed to act in theopposite of the face’s eye gaze; if the frame was red, theinstructions were inverted. The frame shape—a rectangle or adiamond—instructed participants to either perform a button pressor a saccade, respectively. The correct responses for the instructionexamples here illustrated are (1) to press the left button, (2) to lookat the left, (3) to press the right button, and (4) to look at theright. The facial expression images were obtained from the Radboud FacesDatabase (Langner et al.,2010).

Our stimulation sequence was based on three types of visual cues: a facialexpression, a frame shape, and a frame color cue (Fig. 1). The facial expression cues were planned to raise theparticipants’ simultaneous attention to the eyes and mouth (happy and sadfaces looking to the right or the left) to achieve high performance on theproposed task. The combination of emotional expressions and gaze directionsresulted in four different facial cues (Fig.1A): happy left (1), happy right (2), sad left (3), and sad right(4). The information from both the facial expression and frame colour cues(green or red,Fig. 1B) was needed to inferthe direction of action required. If the frame was green, a happy face indicatedto act in the same direction of the face’s eye gaze, and a sad faceindicated to act in the opposite direction of the face’s eye gaze; if theframe was red, the instructions were inverted (i.e., a happy face instructed toact in the opposite direction of the face’s eye gaze). The frame shape(Fig. 1B)—a rectangle or adiamond—instructed participants to either perform a button press or asaccade, respectively. A correct response required the correct interpretation ofthe three cues (facial expression, frame shape, and frame color). Participantswere informed about the task rules through verbal explanation before enteringthe MRI scanner.

**Fig. 2.**
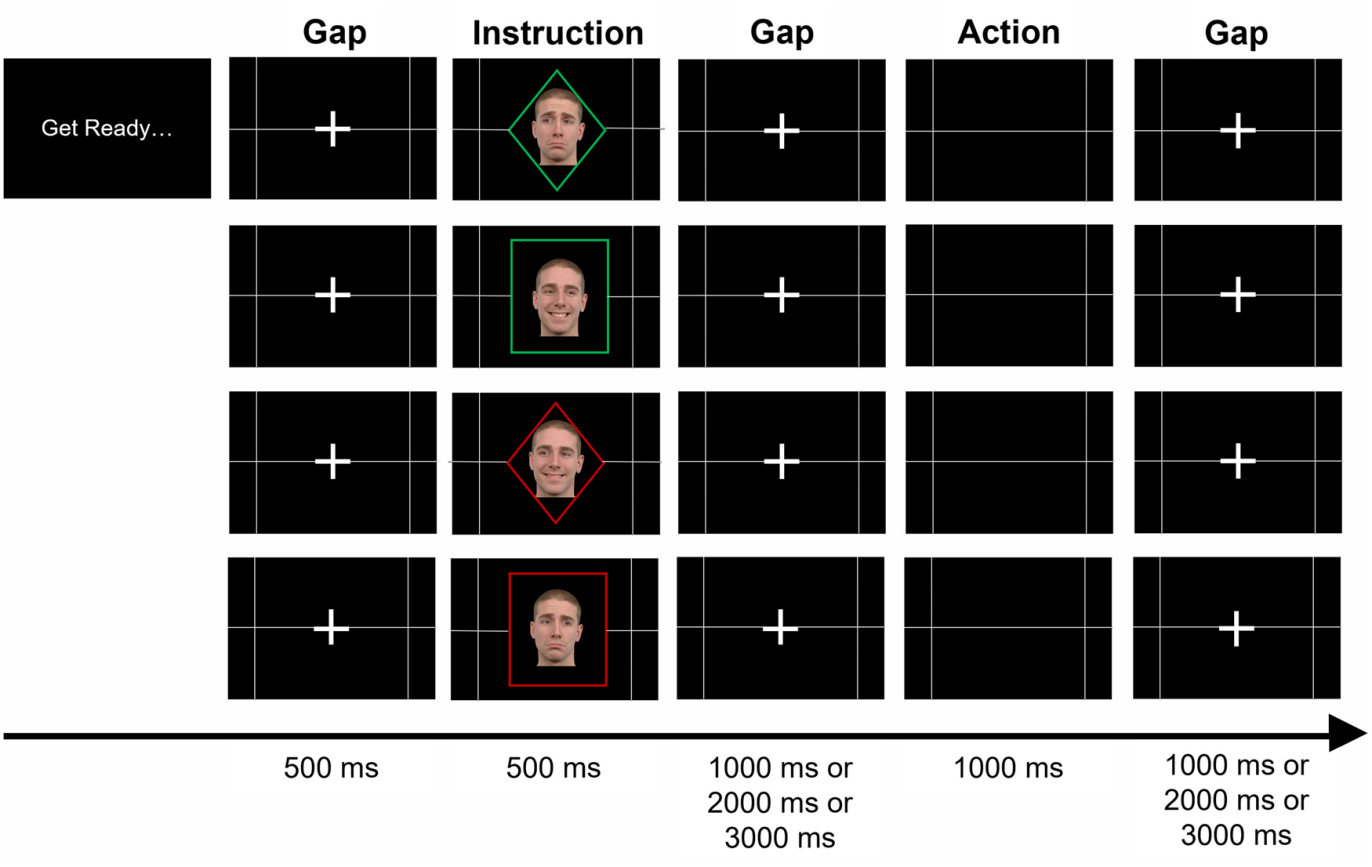
Task design. Each trial sequence comprised the presentation of aninstruction and the response/action moment, which were interleaved withthree gap moments.

The experimental paradigm followed a fast event-related design and included fiveperiods (Fig. 2). Each sequence containedan instruction and action period, and three gaps. Gap periods consisted of ablack background with a white fixation cross in the middle of the screen. Thefirst one occurred before the instruction and lasted for 500 ms. Then, theinstruction was presented during 500 ms. The instruction order was pseudo-randommixing saccade with button press cues and happy with sad faces. The frame colorof the facial cue changed after every three trials. The instruction presentationperiod was followed by a gap of variable duration (1000 ms, 2000 ms, or 3000ms). Participants should then give their response after this when the fixationcross disappeared. They had 1000 ms to perform their button press or saccaderesponse. Lastly, there was another gap period varying from 1000 ms to 3000 ms.We did not provide feedback (see rationale in the Introduction). This paradigmwas adapted fromEstiveira et al. (2022),as it has been previously shown to be an effective approach to studyingerror-monitoring neural circuitry.

Each participant performed 7 experimental runs of 48 trials each. Button pressand saccadic trials, happy and sad, and green and red frame cues werecounterbalanced. Each run included 3 baseline periods that occurred at the endof every 16 trials (white noise with a duration of 10 s, 11 s, or 12 s) andlasted approximately 6 min.

The stimuli were programmed using the Psychophysics Toolbox 3.0 for MATLAB(R2019b) and presented on a 1920 × 1080 resolution monitor with a refreshrate of 60 Hz, 156 cm away from participants. Facial expression images weresized as 6° × 5.20°, while their frames were 10°× 8.6° (height x width, visual angles from the centre toperiphery). The fixation cross had a length of 2.57° ×2.57°. The facial expression images, frames, and fixation cross weredisplayed at the center of the screen. A gray grid designed to help homogenizesaccade behavior included intersections at a horizontal visual angle of19.13° from the center of the screen (participants were instructed tolook at the intersection if they aimed to perform a saccade). The facialexpression images were based on a young adult male and obtained from the RadboudFaces Database (Langner et al.,2010).

### 2.3 Data acquisition

MRI was performed using a 3T Siemens MAGNETOM Prisma Fit scanner (Siemens,Erlangen) using a 64-channel head coil. Functional images were acquired using a2D multi-band (MB) gradient-echo echoplanar imaging (GE-EPI) sequence, with thefollowing parameters: TR/TE = 1000/30.2 ms, voxel size = 2.5× 2.5 × 2.5 mm^3^, 42 axial slices (whole-braincoverage), FOV = 192 × 192 mm^2^, FA = 90°,phase encoding in the anterior-posterior direction, and MB factor = 3. A3D whole-brain anatomical T1-weighted MPRAGE (TR = 2530 ms, TE =3.50 ms, 192 interleaved slices with isotropic voxel size of 1 mm^3^)was collected for subsequent image registration. Respiratory and cardiac datawere recorded at 125 Hz and 500 Hz, respectively, with a respiratory belt andpulse oximeter from the physiological monitoring unit of the MRI system.

Eye-tracking data were recorded using EyeLink 1000 (SR Research, Canada) at asampling rate of 1000 Hz and accuracy of 0.25°–0.5°. Eachrun started with an eye-tracker 9-point calibration and validation session.Button press information (response and response latency) was registered usingMATLAB version R2018b (*.mat).

### 2.4 Behavioral data analysis: participants’ performance

To assess participants’ performance, we analyzed whether their responseswere correct or not. A correct response required an accurate integration of allcues (facial expression, frame color, and frame shape) at the correct timing,that is, during the*Action*period (Fig. 2). Errors included incorrect action direction (e.g.,pressing the right button instead of the left button), incorrect action (e.g.,performing a saccade instead of a button press), omission errors (i.e., notresponding), action direction indecisions (e.g., looking at the right and thenat the left), action indecisions (e.g., looking at the right and then pressingthe right button), simultaneous incorrect action and incorrect action direction,and anticipated errors (including all error types mentioned previously).Anticipated correct responses (i.e., correct responses given before the*Action*period), which corresponded to 13.87 ± 17.12%of all trials, were excluded from further analysis to avoid ambiguity.

Saccades were defined as horizontal movements superior to 4.27° of visualangle (Poletti & Rucci, 2016) fromthe screen center. In addition, a minimum duration of 15 ms was considered toidentify a saccade. We visually inspected some trials of each participant andadapted these parameters for participants with unusual patterns of ocularmovements. The detection of saccadic responses was made in a custom-made MATLABscript (version 2018b, MathWorks).

Participants’ error rate (i.e., the ratio between the erroneous responsesand the sum of correct and erroneous responses) was evaluated according todistinct types of instruction with distinct difficulty levels (happy green,happy red, sad green, and sad red) and learning periods (run 1 to 7). Weconsidered each run a distinct learning period since repeated practice leads tolearning (Toppino & Gerbier,2014).

Additionally, we analyzed how different cues reflected distinct task difficultylevels using a post-fMRI session questionnaire. We asked participants whichcolor cue (green or red) and facial expression cue (happy or sad) were the mostdifficult (with two-choice questions). We measured the percentage ofparticipants that selected each condition.

#### 2.4.1 Behavioral data: statistical analysis

To assess the impact of difficulty and learning period onparticipants’ error rate, we conducted a linear mixed-effects modelwith instruction and run/learning period as factors, and error rate as thedependent variable. We also included the interaction between instruction andrun, but removed fixed factors and interactions without significance tosimplify the model (Seltman, 2018).The participant ID was defined as random variable, and we included a randomintercept for each participant. Results were considered significant for*p*< 0.05. The linear mixed-effects modeling wasperformed using IBM SPSS Statistics 25 software.

### 2.5 fMRI data processing

MRI data were preprocessed using custom scripts in MATLAB (version R2019b,MathWorks), based on the SPM12 software with PhysIO toolbox (Kasper et al., 2017), and FMRIB Software Library(FSL), adapted fromSoares et al. (2022).The pipeline included: (1) slice timing correction; (2) realignment of all fMRIvolumes relative to the first volume; (3) correction of geometric distortionscaused by magnetic field inhomogeneity, with FSL tool TOPUP (Andersson et al., 2003); (4) bias field correction;(5) image registration (functional to structural); (6) estimation of nuisanceregressors (with PhysIO toolbox) such as cardiac and respiratorysignals—RETROICOR regressors (Glover etal., 2000), heart rate variability convolved with the cardiacresponse function, and respiratory volume per time convolved with therespiratory response function—and head motion (6 motion parameters andframewise displacement censoring with threshold = 0.5 mm); (7)segmentation of the T1 structural image (for the CONN standard denoisingpipeline); (8) functional and anatomical images normalization to MontrealNeurological Institute (MNI) space; and (9) spatial smoothing with a 7.5 mmfull-width-at-half-maximum (FWHM) isotropic Gaussian kernel - 3 times the voxelsize (Chen & Calhoun, 2018).

### 2.6 fMRI data analysis

#### 2.6.1 Brain activity analysis

To map the regions involved in error monitoring and learning, we built ageneral linear model (GLM) comprising four regressors for each run:*instruction*,*correct response*,*error*, and*other*(i.e., excludedresponses). The saccade event was set at the end of the horizontal eyemovement, as error-related signals are temporally more similar betweenkeypresses and saccades when the saccade event is defined at the end of theeye movement, probably due to greater impulsiveness during saccadicresponses (Estiveira et al., 2022).The button press event was set in the moment during which the button waspressed. The onset of omission errors was defined based on the mean onsetvalue for the expected response action (saccade or button press) for eachparticipant’s run. The durations were set to zero, except for the*instruction*condition, in which it was set to 500 ms.The design matrix for the GLM included a high-pass filter with a cutoffperiod of 128 s, and regressors were convolved by the canonical hemodynamicresponse function. Cardiac, respiratory, and head motion regressors wereadded to the design matrix as confounding signals.

Group activation maps for the contrast between correct events and errors(performance-based modulation:*error > correct*,computed by*error_R1_+ error_R2_+error_R3_+ error_R4_+error_R5_+ error_R6_+error_R7_> correct_R1_+correct_R2_+ correct_R3_+correct_R4_+ correct_R5_+correct_R6_+ correct_R7_*) and thecontrast between the first two runs’ responses and the last tworuns’ responses (learning-based modulation:*late learningperiod > initial learning period*, computed by*correct_R6_+ error_R6_+correct_R7_+ error_R7_>correct_R1_+ error_R1_+correct_R2_+ error_R2_*) were thengenerated. SPM12 was used to build the GLM and generate the group activationmaps. For visualization purposes, we used BrainNet Viewer (Xia et al., 2013) and MRIcroGL (https://www.nitrc.org/projects/mricrogl).

Moreover, we investigated how the learning period (measured by run number),performance (coded as correct and erroneous responses), and difficulty(assessed by instruction type) interact at the neural response level. Withthat in mind, we extracted the percent of signal change per event at thelevel of our regions of interest (ROIs), the dACC, anterior insula, andputamen. The single-response percent signal change was estimated by dividingthe single-response beta coefficient by the run model intercept(β_0_) (Mayer et al.,2012). The single-response beta coefficient was computed based ona Least-Squares All approach (Abdulrahman& Henson, 2016) using SPM 12. Following this approach,each trial response (correct or erroneous) was modeled as a separateregressor in the GLM. However, for*instruction*and*other*(i.e., excluded responses) conditions, allrepetitions of the same type were collapsed into the same regressor, giventhat, for the statistical analysis, we were only interested in the percentsignal change of*correct response*and*error*conditions.

We used the Brainnetome Atlas (Fan et al.,2016) to functionally define our ROIs, based on which we thenextracted brain response values to include in the linear mixed-effectsmodels analysis. Regarding the error monitoring analysis, we hypothesizedthat the dACC and anterior insula would reveal an increased activationduring errors (compared to correct responses). Note that, in previousstudies, the dACC has also been termed anterior midcingulate cortex (aMCC)(Ninomiya et al., 2018;Ullsperger, 2017;Ullsperger et al., 2014;Vogt, 2016), and the definition of this regionhas been variable (Vogt, 2016).Hereinafter, the dACC is used to refer to the pregenual area 32 (Beckmann et al., 2009;Fan et al., 2016;Palomero-Gallagher et al., 2008). Accordingly, we selected theBrainnetome regions corresponding to the dACC and bilateral anterior insula,which were the pregenual area 32 (A32p) and the conjunction of the ventraland dorsal agranular insula (vIA and dIA). Moreover, we also included thebilateral putamen in our analysis to explore learning-related brain responsemodulation, as suggested by previous literature (Ashby et al., 2010;Baladron & Hamker, 2020;Brovelli et al., 2011;Patterson & Knowlton, 2018;Seger & Spiering, 2011;Tricomi et al., 2009). This ROI wasdefined as the ventromedial putamen (vmPu) Brainnetome region of theatlas.

#### 2.6.2 Functional connectivity analysis

To better understand the brain networks involved in error monitoring andlearning, we performed a generalized psychophysiological interaction (gPPI)connectivity analysis (McLaren et al.,2012) using bivariate regression in the CONN toolbox (Whitfield-Gabrieli & Nieto-Castanon,2012). Preprocessed (but unfiltered—since the CONNstandard denoising pipeline includes filtering) data were imported into thetoolbox, as well as the onsets and durations of the conditions(*baseline*,*instruction*,*correct response*,*error*, and*other*- excluded responses). We then implemented CONNstandard denoising pipeline (Nieto-Castanon,2020). It combines two steps: linear regression to removeconfounding effects in the blood oxygen level-dependent (BOLD) signal andtemporal filtering. Potential confounding effects include noise componentsfrom white matter and cerebrospinal areas, estimated subject-motionparameters, identified outlier scans, constant and first-order linear BOLDtrends, and task effects. Moreover, data were high-pass filtered with acut-off frequency of 0.01 Hz.

To assess the neural networks implicated in error monitoring and learning, weconducted seed-to-voxel analyses for the contrasts*error >correct*and*late learning period > initiallearning period*. The seeds were our ROIs: dACC and bilateralanterior insula for the first contrast, and bilateral putamen for thesecond.

#### 2.6.3 fMRI data: statistical analysis

For the group activation maps concerning the contrast between correct anderroneous responses, as well as between initial and late learning periods,the significance threshold was set at*p*< 0.05 withfamily-wise error (FWE) correction for multiple comparisons. Moreover, tomeasure the impact of learning, performance, and difficulty on the activityof our ROIs (the dACC, anterior insula, and putamen), we conducted a linearmixed-effects model with run/learning, performance, andinstruction/difficulty as factors and single-response percent of signalchange of each ROI as the dependent variable. We also included all possibleinteractions between factors but removed fixed factors and interactionswithout significance to simplify the models (Seltman, 2018). The participant ID was defined as randomvariable, and we included a random intercept for each participant. Usinglinear mixed-effects modeling, we accounted for within and acrosssubjects’ variability and included multiple factors in the analysiswhile considering an unequal number of repetitions (Walker et al., 2019). The linear mixed-effectsmodeling was performed using IBM SPSS Statistics 25 software. Due tomultiple testing (one model for each ROI), we used Bonferroni correction formultiple comparisons by multiplying the*p*-value by thenumber of ROIs. For the seed-to-voxel analyses, the connection threshold wasset at*p*< 0.001 (uncorrected), and the clusterthreshold was set at*p*< 0.01 with FWE correctionfor multiple comparisons.

## 3 Results

### 3.1 Behavioral results: participants’ performance

**Fig. 3.**
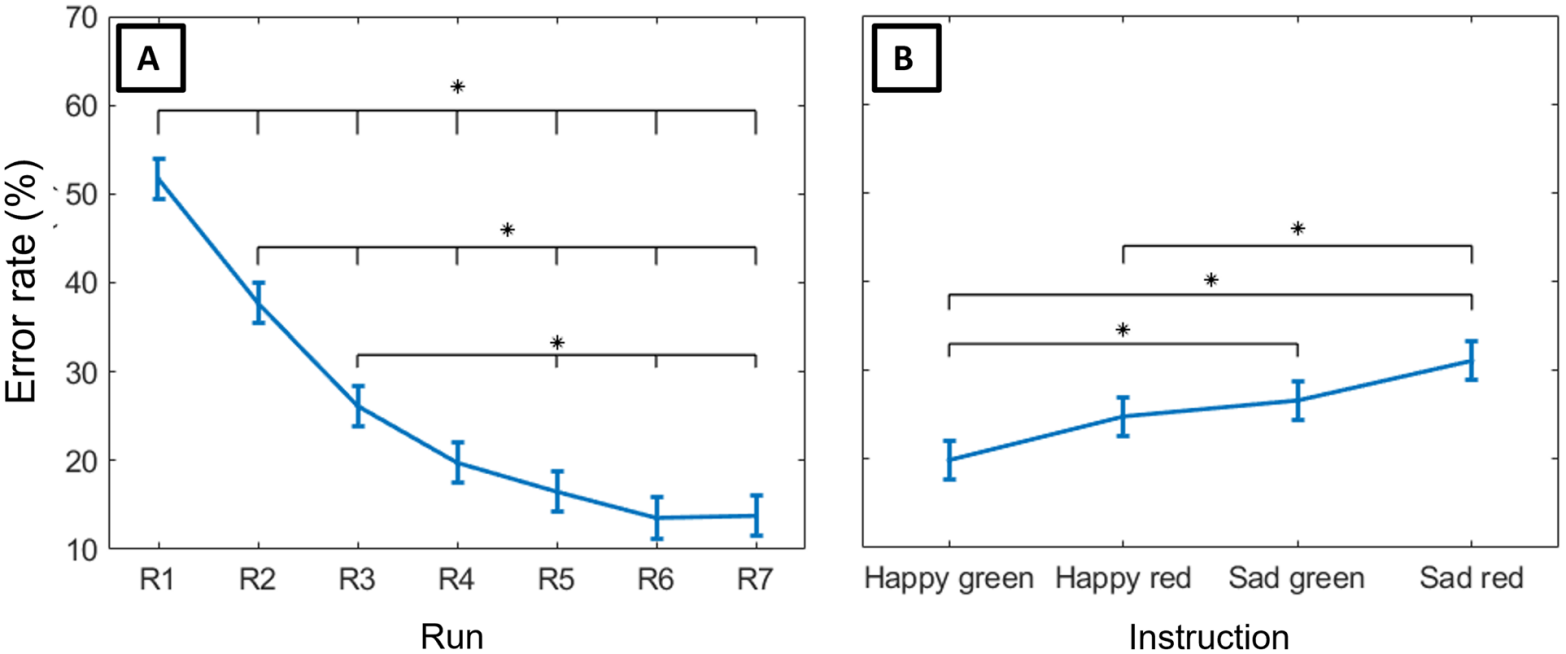
Estimated marginal means of error rate (A) over experimental runs (fromR1 to R7), showing a clear learning curve, and (B) according to eachtype of instruction (*happy green*,*happyred*,*sad green*, and*sadred*). Both factors were found to significantly impact therecorded error rate. Error rate decreased with learning (the error ratein the first two runs was higher than the error rate in the remainingruns, and the error rate in the third run was higher than the error ratein the last three runs) and differed between instructions (fewer errorsoccurred following the*happy green*instruction comparedto the*sad green*and*sad red*instructions, and the number of errors following the*happyred*instruction was lower than the number of errors thatfollowed the*sad red*instruction), revealing distinctdifficulty levels. Error bars represent the standard error of the mean(SEM).

In total, we identified 4604 correct responses and 1210 errors (mean 219.24± 90.27 correct responses and 57.62 ± 35.46 errors perparticipant). A linear mixed-effects model analysis revealed a significantimpact of run (*F*(6, 562.97) = 49.83,*p*= 4.15 × 10^-49^), which is consistent with the learningcurve shown inFigure 3A, and instruction(*F*(3, 563.02) = 9.02,*p*=8.00 × 10^-6^) on error rate (Fig.3B), and no significant interaction between both. To compare theerror rate between all learning periods and instructions, we ran*post-hoc*pairwise comparisons. The error rate in the firsttwo runs was higher than the error rate in the remaining runs, and the errorrate in the third run was higher than the error rate in the last three runs.Moreover, fewer errors occurred following the*happy green*instruction compared to the*sad green*and*sadred*instructions, and the number of errors following the*happy red*instruction was lower than the number of errorsthat followed the*sad red*instruction.Supplementary Table S1details these results.

Regarding the participants’ reports on task difficulty levels, 84.21%considered the*sad*cue more difficult than the*happy*cue, and 89.47% rated the*red*cue asmore demanding than the*green*cue.

### 3.2 Neurophysiological results

#### 3.2.1 Brain activity modulation by error monitoring and learning

**Fig. 4.**
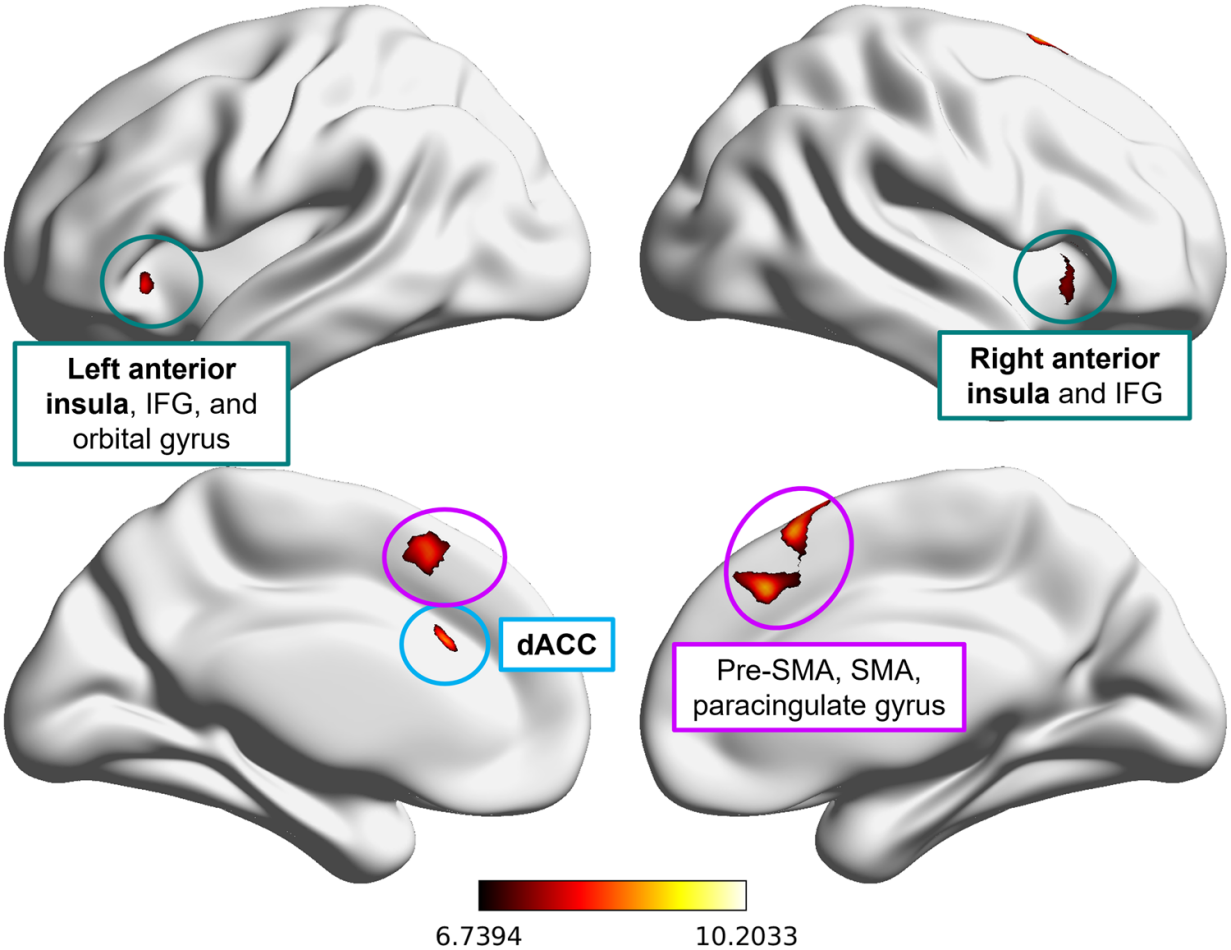
Statistical map of group-level differences in BOLD signal betweencorrect and erroneous responses:*error >correct*,*p*< 0.05 FWE correctedfor multiple comparisons,*k*> 200.Bold-labeled neural regions constitute our ROIs. The colorbar scalerepresents the*t*-values for the contrast.

We started by mapping the regions involved in error monitoring. A groupstatistical map for the contrast between correct responses and errors(*error*>*correct*) was generated(Fig. 4). Clusters identified withsignificant brain activity for this contrast (detailed inSupplementary TableS2) included the dACC, anterior insula, and other regions alsoknown to be involved in error monitoring mechanisms, namely thepre-supplementary motor area (pre-SMA), supplementary motor area (SMA),paracingulate gyrus, inferior frontal gyrus (IFG), and orbital gyrus (Cieslik et al., 2024;Neta et al., 2015;Ninomiya et al., 2018;Ullsperger, 2017;Ullsperger et al., 2014). The statistical map for the contrast*correct*>*error*is shown inSupplementary FigureS1(with details provided inSupplementary TableS2).

**Fig. 5.**
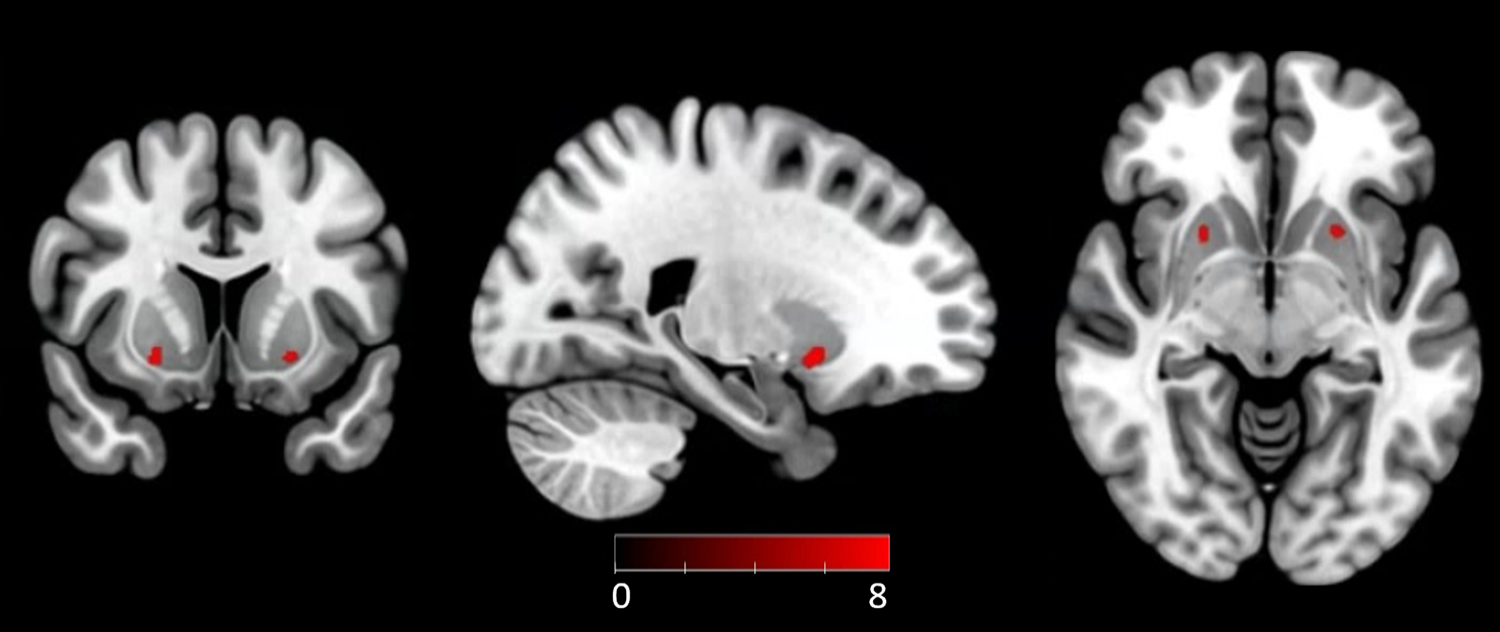
Statistical map of group-level differences in BOLD signal between theinitial and late learning periods (*late learning period> initial learning period*,*p*< 0.05 FWE corrected for multiple comparisons). The colorbarscale represents the*t*-values for the contrast.

Along with error monitoring, we were interested in mapping the regionsinvolved in learning. Therefore, we generated group activation mapsconcerning the contrast*late learning period*>*initial learning period*(Fig. 5). Only the ventral putamen region revealed a significantresponse for this contrast (details provided inSupplementary TableS3). We did not find significant clusters for the contrast*initial learning period*>*late learningperiod*.

#### 3.2.2 Relationship between error monitoring and learning

In addition to the whole-brain contrast statistical maps, we tested theimpact of performance, learning period, and difficulty level on theresponses of our regions of interest, specified in our hypothesis: dACC andanterior insula, and putamen. As mentioned insection 2.6.1, we used the Brainnetome Atlas (Fan et al., 2016) to functionally define ourROIs.SupplementaryFigure S2illustrates the overlay between the clusters thatresulted from the previous activation maps (for the contrasts*error*>*correct*and*late learning period*>*initial learningperiod*) and the Brainnetome Atlas-based ROIs.

The dACC and anterior insula responses showed a similar pattern. A linearmixed-effects modeling analysis revealed a significant effect of performanceon the activity of dACC (*F*(1, 5581.62) = 85.94,*p*-corr. = 7.77 × 10^-20^) andanterior insula (*F*(1, 5149.33) = 141.13,*p*-corr. = 1.19 × 10^-31^). Italso revealed a significant interaction between learning period (run number)and performance on dACC (*F*(12, 5769.74) = 4.38,*p*-corr. = 1.58 × 10^-6^) andanterior insula (*F*(12, 5773.50) = 3.65,*p*-corr. = 5.10 × 10^-5^). Nosignificant effect was found related to task difficulty (measured byinstruction type).

**Fig. 6.**
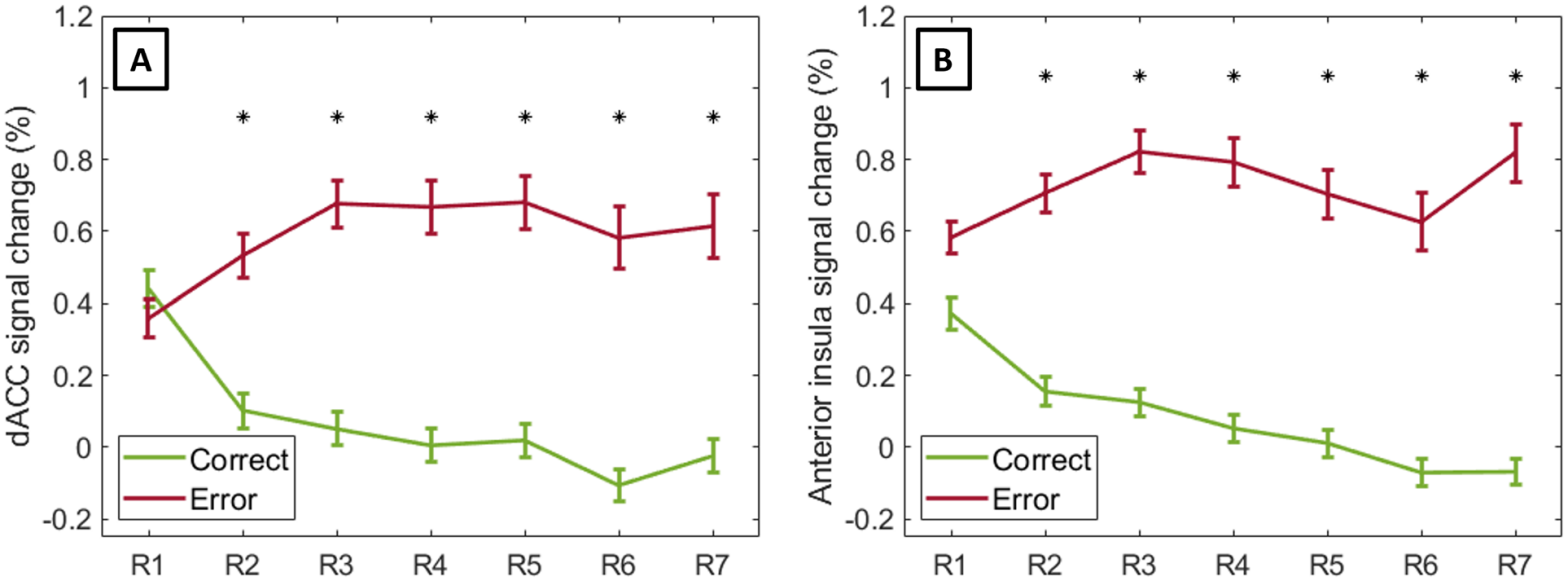
Estimated marginal means of dACC (A) and bilateral anterior insula(B) percent signal change for correct and erroneous responses overlearning periods (here represented as runs 1 to 7— R1 to R7).Error bars represent the SEM. Significant differences(*p*< 0.05 Bonferroni corrected formultiple comparisons) between correct responses and errors aresignalized with an asterisk.

Given that the mixed-effects analyses revealed significant interactionsbetween learning period and performance, we ran*post-hoc*tests to compare the BOLD activity in response to correct trials and errorsper experimental run/learning period (Fig.6). In the first run/learning period, there was no differencebetween correct and erroneous responses for the regions of the saliencenetwork (dACC and anterior insula), but it increased with the progress oflearning: the activity of dACC and anterior insula decreased for correctresponses and increased for errors along the runs. The statistical resultsare detailed in theSupplementary Table S4.

**Fig. 7.**
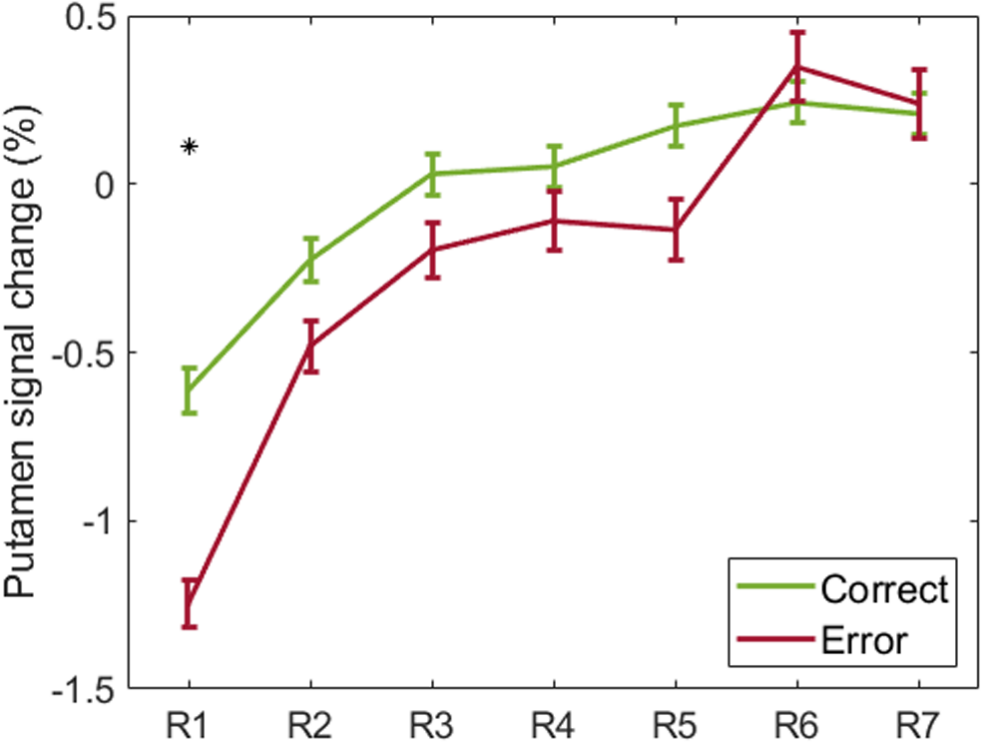
Estimated marginal means of bilateral putamen percent signal changefor correct and erroneous responses over learning periods (hererepresented as runs 1 to 7— R1 to R7). Error bars representthe SEM. Significant differences (*p*< 0.05Bonferroni corrected for multiple comparisons) between correctresponses and errors are signalized with an asterisk.

We also found a significant impact of learning period (*F*(6,5766.21) = 38.04,*p*-corr. = 4.62 ×10^-45^) and performance (*F*(1, 5722.17)= 10.87,*p*-corr. = 0.003) on the activity ofbilateral putamen, as well as a significant interaction between learningperiod and performance (*F*(6, 5769.24) = 2.87,*p*-corr. = 0.024). Given the significantinteraction, we ran*post-hoc*tests to compare the BOLDactivity in response to correct answers and errors for each run/learningperiod (Fig. 7). Putamen activitydiffered between correct and erroneous responses in the first run, but itbecame equivalent between conditions with the effect of learning, featuringan opposite effect to the one seen in the ACC and anterior insula. Theputamen response increased in both cases over time. The statistical resultsare detailed in theSupplementary Table S5.

#### 3.2.3 Neural correlates of error monitoring at the functional connectivity level

**Fig. 8.**
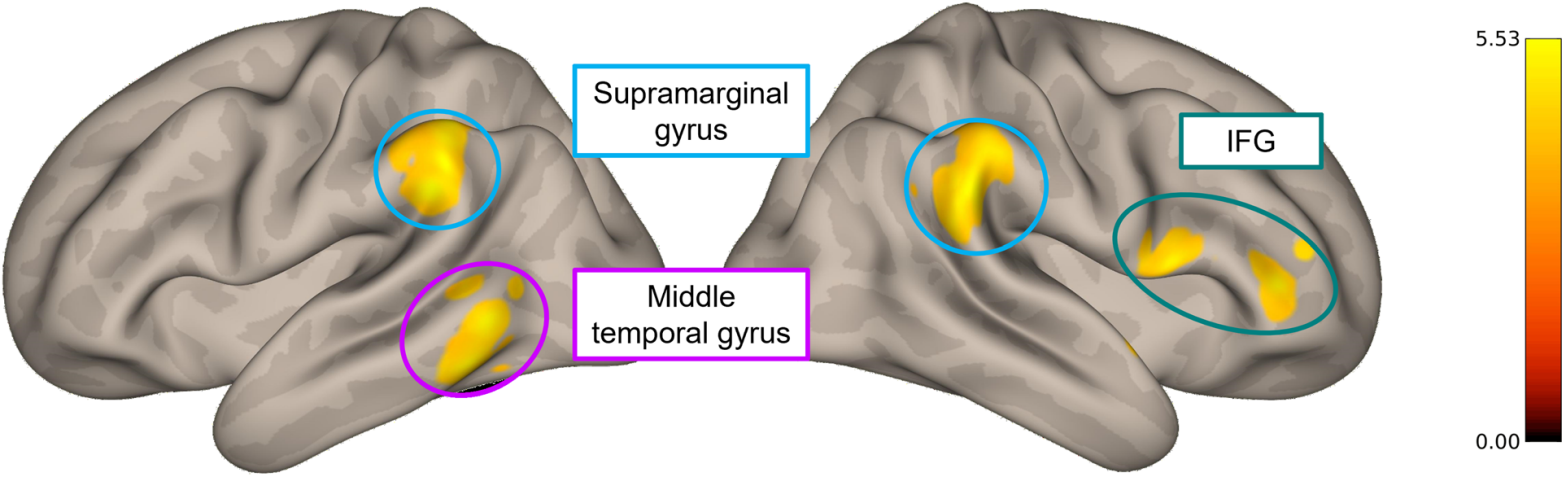
Results from the seed-to-voxel gPPI analysis, using the dACC as seedregion, showing areas of higher functional connectivity in erroneouscompared to correct responses. A voxel threshold of*p*< 0.001 (unc.) was used with a clustersignificance threshold of*p*< 0.01 (FWEcorrected for multiple comparisons). The colorbar scale representsthe*t*-values.

To evaluate the neural networks implicated in error monitoring and learning,we conducted seed-to-voxel analyses for the contrasts*error >correct*(using the dACC and anterior insula as seed regions)and*late learning period > initial learning period*(with the putamen as seed region).Figure8illustrates the results from the seed-to-voxel analysisconcerning the first contrast, using the dACC as seed region, and all thestatistical details are provided inSupplementary Table S6. We did not find significantresults for the remaining analyses.

## 4 Discussion

In this study, we investigated how neural processes of error monitoring interact withlearning when the integration of different facial cues is required. We found thatactivity in the dACC and anterior insula was not modulated by participants’performance in the initial learning period, but increasingly differed betweencorrect and erroneous responses with learning progress. The results showed anopposite pattern for the putamen: its activity was initially modulated by errors,but then similarly increased over time for correct and erroneous responses.

### 4.1 Behavioral results: a clear learning curve and distinct difficulty levels

The performed task led to a well-defined learning curve: in the first run,participants had a mean error rate of above 50%, and in the last, of less than15%. Additionally, the different types of conditions led to distinct errorrates, confirming the existence of different difficulty levels, as alsoperceived and reported by the participants. There are few error monitoringstudies with clear learning curves (Daniel& Pollmann, 2012;Maurer etal., 2019;McDougle, 2022),which reveals that this task is appropriate to study the relationship betweenperformance monitoring and learning while accounting for potential difficultyeffects. It was relevant to measure the influence of difficulty on our results,and to disentangle the effects of error monitoring and task difficulty, sinceprevious literature proposed that the dACC is modulated by choice difficulty(Shenhav et al., 2014).

### 4.2 Differences between correct responses and errors in key performance monitoring regions increase with learning

As hypothesized, the dACC and anterior insula showed increased BOLD signal duringerroneous than correct responses (although only after an initial learningperiod). These two regions have been described as key regions of the errormonitoring neural circuitry and linked to error awareness (Dali et al., 2023;Orr & Hester, 2012). The dACC has been hypothesized to signalthe need for additional cognitive control resources following errors (Heilbronner & Hayden, 2016;Weiss et al., 2018).

When assessing how the dACC response to error changed with the learning progress,we found that, at the beginning of the task, the response to correct anderroneous events was similar, and, over time, the differences between theseconditions became more evident. Along the learning periods, for correctresponses, the dACC activity decreased; for errors, it increased.Kelly and Garavan (2005)have argued thatdecreases in the extent or intensity of activations are expected with taskpractice due to increased neural efficiency. Activation in attentional andcontrol regions, for instance, may return to baseline on account of practice.Matsumoto et al. (2007), usingintracranial recordings in monkeys, demonstrated that a subgroup of ACC neuronsresponds to positive visual feedback, and the magnitude of these responsesdecreases throughout adaptation. The authors reported that bigger responses wereevoked by positive feedback when the monkey was not confident with its action,that is, when the feedback was not predicted (and thus useful for learning).Here, on one hand, the dACC activity in correct trials decreased with practice,which may be related to increased neural efficiency and/or task confidence, dueto learning. On the other hand, the dACC increased with time in response toerrors, which may be related to the fact that, as learning proceeds, erroneousresponses become more unexpected. In the first run, errors are not surprisingsince participants are still learning the task, but with practice erroneousresponses become uncommon.

These results are compatible with the predicted response outcome (PRO) model(Alexander & Brown, 2011),according to which the ACC and surrounding medial prefrontal cortex predict thelikelihood of future outcomes and compare it with the actual outcome. When theexpected and actual outcomes do not match, a reward prediction error is producedand communicated to the prediction units. The predictions are then updated forfuture reference. Therefore, according to this theory, the ACC is primarilysensitive to the unpredictability of outcomes. The reinforcement learning model(Holroyd & Coles, 2002,^2008^) may also explain the increase in dACCactivity during erroneous responses with learning and practice, as it similarlysuggests that the dACC activity reflects reward prediction errors. This theoryproposes that the dACC selects the action plan for a particular task based onreward prediction error signals carried to the dACC by the midbrain dopaminergicsystem (with massive projections to the striatum) (Daniel & Pollmann, 2014).

The response to error in the anterior insula had similar progress as the dACC, inline with previous studies reporting co-activation of these neural regions aspart of the salience network (Harsay et al.,2012;Klein et al., 2007;Ullsperger et al., 2010). Theanterior insula has mainly been linked to error awareness (Dali et al., 2023;Harsay et al., 2012;Klein et al.,2013), suggesting that participants became more aware of theirmistakes with learning. Moreover, the anterior insula has been previously linkedto learning (Horing & Büchel,2022;Kornhuber et al., 1995;Palminteri et al., 2012). Forinstance,Palminteri et al. (2012)demonstrated that damage to the anterior insula impairs punishment avoidance andhence affects the learning process.

Additionally, we evaluated the impact of task difficulty level (measured by thedifferent instruction types) on the dACC and anterior insula activity duringerror monitoring progress with learning but did not find significant results.This finding is at odds with theories of dACC function that suggest that thedACC codes for choice difficulty (Shenhav etal., 2014). The dACC functions are still a matter of debate, andseveral models have been proposed and discussed in the literature (Vassena et al., 2017). Some of themaccommodate our findings—for instance, the PRO model (Alexander & Brown, 2011) and the reinforcementlearning model (Holroyd & Coles,2002,^2008^) —but notthe one proposed byShenhav et al.(2014).

### 4.3 The role of putamen in learning from errors

The putamen revealed to have increasing BOLD activity over time during our task.Its response progressed differently from the dACC and anterior insula: itdiffered between correct and erroneous responses in the beginning and thensimilarly increased for both correct and erroneous responses with learning.

According to the reinforcement learning theory, the reward prediction error iscommunicated to the ventral striatum (including the ventral putamen), where itis used to improve outcome predictions and compare them to actual outcomes(Daniel & Pollmann, 2014;Ullsperger et al., 2014). This mayhappen during the initial learning periods, leading to differences in putamenactivity between correct and erroneous responses, as found in our results. Withlearning, this differentiation is taken over by the dACC and anterior insula forperformance monitoring. Similarly, Daniel et al. (2012) demonstrated increasedactivation for correct than erroneous responses only before extensive training.Afterward, the anterior insula, medial prefrontal areas, and midbrain revealedhigher activity for errors than correct answers.

The putamen has been linked to habit learning and automaticity (Ashby et al., 2010;Baladron & Hamker, 2020;Brovelli et al., 2011;Miyachi etal., 1997,^2002^;Patterson & Knowlton, 2018;Reading et al., 1991;Seger & Spiering, 2011;Tricomi et al., 2009;Yin et al., 2006,^2004^),which is consistent with the increase in putamen response with learning progressfound in our results. While habit formation is sensitive to changes in outcomevalue, actions become more automatic with practice and, if a stimulus-responsehabit has been formed, a cue might evoke its associated response despite theoutcome (Dickinson & Balleine,2002;Tricomi et al., 2009).This may explain the difference in putamen activity between correct anderroneous responses found at the beginning of our session only.

### 4.4 Other regions modulated by errors

In addition to these regions, the paracingulate gyrus, pre-SMA, SMA, IFG, andorbital gyrus revealed an increased activity during errors compared to correctresponses. Previous studies have shown that adjacent areas to the dACC, such asthe paracingulate gyrus, pre-SMA, and SMA, are also modulated during responseerrors, conflict, negative feedback, and surprise (Neta et al., 2015;Ullsperger, 2017). The pre-SMA is one of the most reported regionsin this regard (Fu et al, 2023;Ninomiya et al., 2018;Ullsperger, 2017;Ullsperger et al., 2014). Some studies suggested that the pre-SMAactivity is mainly linked to conflict monitoring and showed a spatialdissociation between conflict and error monitoring, located in the pre-SMA anddACC, respectively (Garavan et al., 2003;Ullsperger & Von Cramon,2001). Others suggested that the neural activation in response to errorslocated in the dACC extends to the pre-SMA (Iannaccone et al., 2015;Jahn etal., 2016).Fu et al. (2023)have described the existence of two frontal regions with errormonitoring-related activity: the supplementary motor complex, within thesuperior frontal gyrus (SFG) and including the SMA, pre-SMA, and supplementaryeye field; and the ACC. Moreover, the pre-SMA and IFG, which seem involved inresponse inhibition, have also been related to post-error behavior adjustments,namely to post-error slowing (Ullsperger,2017). The orbitofrontal cortex, in turn, appears to encode outcomevalence, outcome expectancies, and information about rewards and choices (Ullsperger et al., 2014).

### 4.5 How do error-related and learning-related brain regions communicate?

To clarify how error-related and learning-related neural regions interact duringerrors, we conducted seed-to-voxel analyses that revealed augmented functionalconnectivity during errors (compared to correct responses) between the dACC andthe IFG, supramarginal gyrus, and middle temporal gyrus. These regions have beenpreviously linked to error monitoring (Barke etal., 2017;Cieslik et al.,2024;Dali et al., 2023;Gauvin et al., 2016;Neta et al., 2015;Ullsperger et al., 2014), andDu etal. (2020)have also shown that the dACC has strong functionalconnectivity with the IFG and supramarginal gyrus.

The IFG has been linked to inhibitory control (Aron et al., 2004,^2007^;Ullsperger et al., 2014), and previousliterature has suggested that the middle temporal gyrus is associated with theprocessing of awareness of discrepancies (VanKemenade et al., 2019). Besides, the supramarginal gyrus has beenproposed to integrate a system that detects and direct attention to relevantstimuli during periods of internally focused mental activity (Corbetta & Shulman, 2002;Reniers et al., 2012). We suggest that, when an erroris committed, the middle temporal gyrus detects the discrepancy between theexpected and obtained outcome, the supramarginal gyrus directs attention to theerroneous action, and the dACC signals the error to other regions to triggeradjustments and optimize behavior, namely to the IFG, that inhibits futuremistakes.

Apart from the IFG, we did not find these regions (the middle temporal gyrus andsupramarginal gyrus) in the group map for the contrast between correct responsesand errors, due to the differences between the GLM and gPPI analyses (Satake et al., 2024). While the GLManalysis detects the BOLD response related to neural activation during the taskcompared with the resting period, the gPPI functional connectivity analysisevaluates the synchronization of neuronal fluctuations influenced by the task.Gerchen and Kirsch (2017)demonstrated that task-evoked activation and connectivity effects reflectseparable and complementary information.

Interestingly, only the dACC showed regions of significantly higher connectivityfollowing errors compared to correct responses, suggesting a functionaldifferentiation between the dACC and anterior insula. This distinction had beenpreviously proposed byHarsay et al.(2012), who argued that the anterior insula is more specialized inprocessing multimodal sensory input, whereas the dACC, which is connected toaction selection and execution systems, triggers adaptive actions in response tothis input (Estiveira et al., 2022).

### 4.6 Limitations and future work

The main limitation of our study is the sample size (n = 21); therefore,future research should replicate this work. Moreover, the clusters we obtainedfrom the statistical maps concerning the contrasts between, on the one hand,correct and erroneous responses and, on the other, initial and late learningperiods, were smaller than the ROIs defined based on the Brainnetome atlas. Inthe future, we aim to study the functional parcellation of our ROIs during errormonitoring.

Given that detecting, processing, and signaling errors is vital for regulatingbehavior, preventing future mistakes, and learning, less efficient errormonitoring mechanisms may lead to difficulties in everyday life (Goldberg et al., 2011;Hüpen et al., 2016). Therefore, it would berelevant to study the evolution of error-related neural processes throughoutlearning in populations with altered error monitoring mechanisms, such asautism, attention-deficit/hyperactivity disorder, and obsessive-compulsivedisorder (Bellato et al., 2021).

## 5 Conclusions

In this study, we aimed to understand how neural processes of error monitoring relateto the learning progress. Our findings provide evidence for the interplay betweenerror monitoring and learning neural processes. The activity in the dACC, anteriorinsula, and putamen was found to be modulated by the interaction between performancemonitoring and learning.

The modulation of dACC and anterior insula response by errors had a similar evolutionwith learning: in the beginning, the neural responses for correct events and errordid not differ but, with learning, the differences became evident, with asimultaneous decrease in correct responses and increase following errors. This seemsto be related to prediction error progress with learning. Importantly, our resultsalso revealed two learning phases that modulate the putamen activity. During theinitial phase—habit formation through trial-and-error (reinforcement)learning—, the putamen activity following correct and erroneous responses wasdistinct. Its activation increased with practice-based learning, and, during thesecond phase—habit consolidation—, actions became more automatic withpractice (and insensitive to changes in outcome value).

We found a temporal distinction in error-related neural responses between the putamen(which showed an explicit relation with the learning curve) and the dACC/anteriorinsula. Correct and erroneous responses were differentiated in the putamen in theinitial learning period, and later in the dACC and anterior insula, suggesting achronometric relationship between circuits underlying learning and error monitoringin the basal ganglia and salience network.

## Supplementary Material

Supplementary Material

## Data Availability

The dataset used here will be made available once the project, within which thisstudy is integrated, is completed. The group activation and connectivity maps areavailable athttps://doi.org/10.5281/zenodo.13742605, and all the code used forpre-processing and analysis is available athttps://github.com/CIBIT-UC/errormonitoring_learning.git.
