# Supplementary material for: A chronometric relationship between circuits underlying learning and error monitoring in the basal ganglia and salience network
Source: Imaging Neurosci (Camb). 2024 Nov 5;2:imag-2-00343. doi: 10.1162/imag_a_00343 (PMC12290811; doi:10.1162/imag_a_00343)
Supplement: Supplementary Material [file imag_a_00343-supp.pdf]

Supplementary material

Table S1. A mixed-effects analysis revealed a significant effect of the run/learning period and instruction/difficulty on the error rate (%). We ran *post-hoc* tests to compare the error rate between all learning periods and all instructions. Significant differences (p < 0.05) are signalized with an asterisk and highlighted with bold lettering. *Post-hoc* tests were Bonferroni corrected for multiple comparisons.

Error rate			
**Parameter**	**Estimate**	***p***	**Effect size**
<u>Learning period:</u>			
**Run 1 – Run 2**	14.01 ± 2.86	2.80×10^{-5}*	1.07
**Run 1 – Run 3**	25.55 ± 2.87	1.70×10^{-16}*	1.94
**Run 1 – Run 4**	31.96 ± 2.86	6.23×10^{-25}*	2.44
**Run 1 – Run 5**	35.23 ± 2.87	1.82×10^{-29}*	2.68
**Run 1 – Run 6**	38.18 ± 2.87	6.51×10^{-34}*	2.90
**Run 1 – Run 7**	37.94 ± 2.87	1.49×10^{-33}*	2.88
**Run 2 – Run 3**	11.54 ± 2.87	0.001*	0.88
**Run 2 – Run 4**	17.95 ± 2.86	1.54×10^{-8}*	1.37
**Run 2 – Run 5**	21.22 ± 2.87	1.18×10^{-11}*	1.61
**Run 2 – Run 6**	24.17 ± 2.87	7.05×10^{-15}*	1.84
**Run 2 – Run 7**	23.94 ± 2.87	1.30×10^{-14}*	1.82
Run 3 – Run 4	6.41 ± 2.87	0.549	0.49
**Run 3 – Run 5**	9.67 ± 2.88	0.018*	0.73
**Run 3 – Run 6**	12.63 ± 2.88	2.95×10^{-4}*	0.96
**Run 3 – Run 7**	12.39 ± 2.88	4.22×10^{-4}*	0.94
Run 4 – Run 5	3.27 ± 2.87	1.000	0.25
Run 4 – Run 6	6.22 ± 2.87	0.648	0.47
Run 4 – Run 7	5.99 ± 2.87	0.792	0.46
Run 5 – Run 6	2.95 ± 2.88	1.000	0.22
Run 5 – Run 7	2.72 ± 2.88	1.000	0.21
Run 6 – Run 7	-0.23 ± 2.88	1.000	-0.02
<u>Instruction</u>:			
**Sad red – Happy green**	11.21 ± 2.18	2.00×10^{-6}*	1.12
**Sad red – Happy red**	6.28 ± 2.17	0.023*	0.63
Sad red – Sad green	4.50 ± 2.17	0.232	0.45
**Sad green – Happy green**	6.72 ± 2.18	0.013*	0.67
Sad green – Happy red	1.80 ± 2.17	1.000	0.18
Happy red – Happy green	4.92 ± 2.18	0.147	0.49

Table S2: Regions that showed significantly greater BOLD signal for erroneous than correct responses (*error* > *correct*, $p < 0.05$, FWE-corr., $k > 200$), and for correct than erroneous responses (*correct* > *error*, $p < 0.05$, FWE-corr.).

**Significant clusters: *error* > *correct***					
**Peak voxel (MNI)**			**Region**	***T***	**Cluster size (voxels)**
x	***y***	***Z***			
	Cluster 1				
3	30	40	Paracingulate gyrus R	10.20	
-2	16	52	Pre-SMA L	9.81	
8	22	57	Pre-SMA R	9.13	
-6	26	26	dACC L	9.03	2236
12	12	64	SMA R	9.01	
10	20	64	Pre-SMA R	8.81	
0	21	39	Paracingulate gyrus L	8.27	
	Cluster 2				
-46	18	3	IFG L	8.59	
-36	26	-4	Orbital gyrus L	5.34	422
-38	22	8	IFG L	5.31	
-28	20	-8	Anterior insula L	5.03	
-27	20	-16	Orbital gyrus L	4.84	
	Cluster 3				
42	22	8	IFG R	7.88	
33	21	-2	Anterior insula R	7.66	268
33	21	-8	Anterior insula R	7.45	
45	16	-6	Anterior insula R	7.34	
**Significant clusters: *correct* > *error***					
**Peak voxel (MNI)**			**Region**	***T***	**Cluster size (voxels)**
x	***y***	***z***			
9	-21	68	SMA R	8.90	6
-26	-3	-9	Putamen L	8.18	37

Table S3: Regions that showed significantly greater BOLD signal for the late than the initial learning period (*late learning period* > *initial learning period,* $p < 0.05$, FWE-corr.).

**Significant clusters:** ***late learning period > initial learning period***					
**Peak voxel (MNI)**			**Region**	***T***	**Cluster size (voxels)**
x	***y***	***Z***			
-22	12	-9	Putamen L	8.00	24
22	10	-9	Putamen R	7.62	34

Table S4: Results of the mixed-effects analyses performed to study the effect of run/learning period, performance, and instruction/difficulty on the activity of dACC and anterior insula. We removed factors and interactions without a significant fixed effect and reran the mixed effect analyses. The estimates of fixed effects for the significant factors or interactions are shown. For the significant interaction between run/learning period and performance, we ran *post-hoc* tests to compare neural activity between correct and erroneous responses during each run. Significant differences ($p < 0.05$) are signalized with an asterisk and highlighted with bold lettering. *Post-hoc* tests were Bonferroni corrected for multiple comparisons and for multiple ROIs.

**dACC**				
**Parameter**	**Estimate**	***p***[a]	***p*-corr.**[a,b]	**Effect size**
Learning period × Performance:				
Error – Correct (Run 1)	-0.08 ± 0.11	0.441		-0.16
**Error – Correct (Run 2)**	0.43 ± 0.11	1.62×10^{-4}	4.86×10^{-4}*	0.85
**Error – Correct (Run 3)**	0.63 ± 0.12	4.13×10^{-7}	1.24×10^{-6}*	1.15
**Error – Correct (Run 4)**	0.66 ± 0.14	2.00×10^{-6}	6.00×10^{-6}*	1.03
**Error – Correct (Run 5)**	0.66 ± 0.14	3.00×10^{-6}	9.00×10^{-6}*	1.03
**Error – Correct (Run 6)**	0.69 ± 0.17	3.50×10^{-5}	1.05×10^{-4}*	0.89
**Error – Correct (Run 7)**	0.64 ± 0.17	1.27×10^{-4}	3.81×10^{-4}*	0.82
**Anterior insula**				
**Parameter**	**Estimate**	***p***[a]	***p*-corr.**[a,b]	**Effect size**
Learning period × Performance:				
Error – Correct (Run 1)	0.21 ± 0.10	0.042	0.126	0.46
**Error – Correct (Run 2)**	0.55 ± 0.11	5.57×10^{-7}	1.67×10^{-6}*	1.09
**Error – Correct (Run 3)**	0.70 ± 0.12	5.97×10^{-9}	1.79×10^{-8}*	1.27
**Error – Correct (Run 4)**	0.74 ± 0.13	3.15×10^{-8}	9.45×10^{-8}*	1.24
**Error – Correct (Run 5)**	0.69 ± 0.14	3.93×10^{-7}	1.18×10^{-6}*	1.08
**Error – Correct (Run 6)**	0.70 ± 0.16	1.5×10^{-5}	4.50×10^{-5}*	0.95
**Error – Correct (Run 7)**	0.89 ± 0.16	3.88×10^{-8}	1.16×10^{-7}*	1.21

a) Bonferroni corrected for multiple comparisons.

b) Bonferroni corrected for multiple ROIs.

Table S5: Results of the mixed-effects analyses performed to study the effect of run/learning period, performance, and instruction/difficulty on the activity of bilateral putamen. We removed factors and interactions without a significant fixed effect and reran the mixed effect analyses. The estimates of fixed effects for the significant factors or interactions are shown. For the significant interaction between run/learning period and performance, we ran *post-hoc* tests to compare putamen activity between correct and erroneous responses during each run. Significant differences (p < 0.05) are signalized with an asterisk and highlighted with bold lettering. *Post-hoc* tests were Bonferroni corrected for multiple comparisons and for multiple ROIs.

**Putamen**				
**Parameter**	**Estimate**	***p***[a]	***p*-corr.**[a,b]	**Effect size**
Learning period × Performance:				
**Error – Correct (Run 1)**	-0.63 ± 0.12	1.34 × 10^{-7}	4.02 × 10^{-7}*	-1.15
Error – Correct (Run 2)	-0.26 ± 0.13	0.045		-0.44
Error – Correct (Run 3)	-0.23 ± 0.14	0.103		-0.36
Error – Correct (Run 4)	-0.16 ± 0.16	0.296		-0.22
Error – Correct (Run 5)	-0.31 ± 0.16	0.051		-0.42
Error – Correct (Run 6)	0.11 ± 0.19	0.567		0.13
Error – Correct (Run 7)	0.03 ± 0.19	0.869		0.03

a) Bonferroni corrected for multiple comparisons.

b) Bonferroni corrected for multiple ROIs.

Table S6: Regions of increased connectivity with the dACC following errors compared to correct responses. These clusters resulted from a seed-to-voxel gPPI analysis with a voxel threshold of $p < 0.001$ (unc.) and a cluster threshold of $p < 0.01$ (FWE corr.).

**Significant clusters: *error* > *correct* (seed-to-voxel analysis)**					
**Peak voxel**			**Region**	***T***	**Cluster size**
x	***y***	***z***			
-52	-56	2	Middle temporal gyrus L	6.96	418
62	-34	32	Supramarginal gyrus R	6.30	520
56	12	4	IFG R	6.15	171
50	40	4	IFG R	5.77	153
-58	-42	36	Supramarginal gyrus L	5.59	511

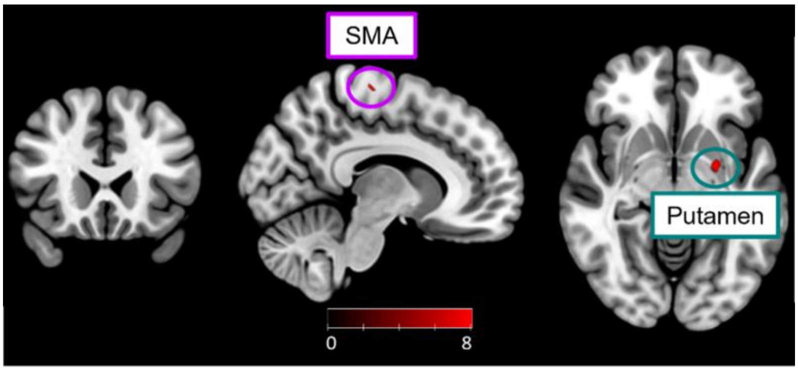

Figure S1: Statistical map of group-level differences in BOLD signal between correct and erroneous responses: *correct* > *error*, $p < 0.05$ FWE corrected for multiple comparisons. The colorbar scale represents the *t*-values for the contrast.

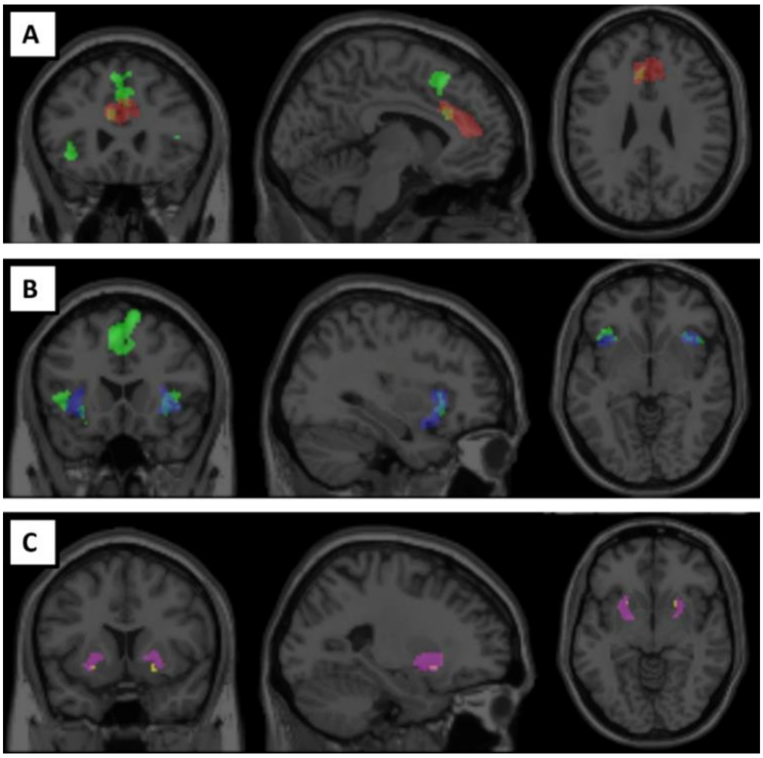

Figure S2: Overlay between the clusters that resulted from the activation maps and the Brainnetome Atlas-based ROIs. A) Overlay between the dACC atlas-based ROI (in red) and the activation map of group-level differences in BOLD signal between correct and erroneous responses (in green: *error* > *correct*, $p < 0.05$ FWE corrected for multiple comparisons, $k > 200$). B) Overlay between the anterior insula atlas-based ROI (in blue) and the activation map of group-level differences in BOLD signal between correct and erroneous responses (in green: *error* > *correct*, $p < 0.05$ FWE corrected for multiple comparisons, $k > 200$). C) Overlay between the ventromedial putamen atlas-based ROI (in purple) and the activation map of group-level differences in BOLD signal between the initial and late learning periods (in yellow: *late learning period* > *initial learning period*, $p < 0.05$ FWE corrected for multiple comparisons).
